# A Web App for the Animal Culture Database and a Template for Deploying Comparative Trait Databases With Shiny

**DOI:** 10.1002/ece3.71913

**Published:** 2025-08-19

**Authors:** Kiran Basava, Cristian Román‐Palacios

**Affiliations:** ^1^ College of Information Science University of Arizona Tucson Arizona USA

**Keywords:** database, open source, Shiny, template

## Abstract

There is a large and growing number of trait databases in ecology and evolution. Structured and open‐access data repositories are important resources for allowing researchers to explore, visualize, and download such data for analysis. In this paper, we detail the design and structure of a simple Shiny web app intended to deploy trait databases as interactive web platforms. Using the Animal Culture Database (ACDB) as a case study, we highlight the web app's functionality and potential transversal applications for research and deploying other databases. The ACDB is a resource compiling socially transmitted traditions across the animal tree of life. It integrates multiple linked tables with explicit geospatial information, enabling data visualization and analysis of cultural behaviors across diverse taxa. The web app, accessible at https://datadiversitylab.github.io/ACDB/, provides users with an intuitive interface to explore the latest version of the database, including population‐level data, behavioral descriptions, and geographic distributions. The code to deploy this web app, and a simplified version to deploy a basic template version, are available on the GitHub repositories https://github.com/datadiversitylab/ACDB and https://github.com/datadiversitylab/generic_shiny_data respectively.

## Graphical abstract

We introduce a Shiny web application that deploys the Animal Culture Database (ACDB) as an interactive platform. The ACDB is a relational database compiling socially transmitted traditions across the animal tree of life. We present the code to deploy this database as a web app available at https://github.com/datadiversitylab/ACDB, as well as code for a simplified template available at https://github.com/datadiversitylab/generic_shiny_data, to be cloned and used to deploy trait databases for other studies.

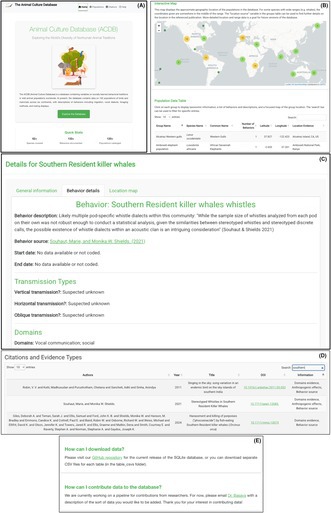

## 1 Introduction

Data‐driven research is fundamental to ecology and evolutionary biology, informing conservation strategies and understanding of species interactions and ecological and macroevolutionary patterns (Farley et al. 2018; Kokol et al. 2022; Todman et al. 2023). As ecological and evolutionary datasets grow in scale and complexity (Farley et al. 2018; McCrea et al. 2023), the need for structured and accessible data repositories becomes increasingly crucial (Bach et al. 2012; Schneider et al. 2019). While datasets can be spatial, hierarchical, or network‐based, many of them are simply tabular, encompassing structured observations, species traits, and experimental results (e.g., Todman et al. 2023; Vanderbilt et al. 2022). Traditionally, tabular data is shared in Microsoft Excel (XLS or XLSX) or comma‐separated values (CSV) formats, either as Supporting Information in publications or on static repositories (Herold 2015; Kambouris et al. 2024; Whitlock 2011). Although this approach generally ensures long‐term availability, it also limits interactivity, discoverability, and efficient data querying (Anderson et al. 2006; Augustine et al. 2024; Gomes 2025; Pinto Padilla and Griffin 2023; Roche et al. 2015). As a result, many datasets remain underutilized due to difficulties in searching and integrating them into existing or newly developed workflows.

Here, we introduce a simple web interface for deploying tabular datasets in an interactive, user‐friendly manner. We use the Animal Culture Database (ACDB) as an example. The ACDB, introduced in a previous paper (Basava et al. 2025), compiles cultural behaviors documented among wild animal populations globally into a relational database. Researchers have documented multiple ways that cultural behaviors affect species' abilities to respond to climate change and human disturbances to the environment, with habitat fragmentation and biodiversity loss threatening existing behavioral traditions even as social learning abilities allow for some populations to adapt to changing environments (Brakes et al. 2019; Gruber et al. 2019; Keith and Bull 2017; Wooster et al. 2024). The ACDB was created in response to calls to integrate this knowledge of animal culture into conservation strategies (Brakes et al. 2019; Brakes et al. 2025), and currently includes variables on cultural behaviors for 120 populations of 62 species. To enhance accessibility and usability of the ACDB, we developed an interactive web application using Shiny (Chang et al. 2024). The web app, presented in this paper, allows users to explore population‐level data, behavioral descriptions, and geographic distributions while linking behaviors to evidence for social transmission pathways.

Web‐based access to datasets enhances accessibility, interactivity, and reproducibility by allowing users to filter, visualize, and download data subsets in real time (e.g., Jentsch et al. 2024; Pinto Padilla and Griffin 2023; Rodríguez‐Moreno et al. 2024). Enabling web access also provides a structured approach to metadata documentation and reduces redundancy by streamlining comparative analyses. Several frameworks exist for web‐based data release, such as Open Data Kit (ODK, Brunette et al. 2013), DataONE (Allard 2012), and Cross‐Linguistic Data Formats (CLDF, Forkel et al. 2018). However, these platforms often require specialized knowledge for implementation and are tailored to specific data types, limiting adaptability for general tabular datasets. Shiny offers a flexible alternative, enabling interactive data exploration, visualization, and download functionalities without extensive web development expertise. Although several datasets have been deployed using Shiny (e.g., MacaqueNet, De Moor et al. 2023; ZooTraits, Gonçalves‐Souza et al. 2024), to our knowledge, no simple and standardized template exists for structuring and deploying datasets via Shiny, leading to inconsistent implementation across projects. Through the release of this Shiny app as open source and licensed under MIT (German and González‐Barahona 2009; Saltzer 2020), we further provide a scalable and reusable template for potential dataset deployment, aimed at supporting broader efforts to enhance data accessibility and transparency in ecology and evolutionary biology.

In the following sections, we first introduce the use of the template for deploying the Animal Culture Database (ACDB), a relational database, as our selected case study with Shiny. Second, we outline the current structure of the app for the ACDB. Third, we discuss approaches to extend the web app template used to deploy the ACDB. Finally, we provide a brief overview of the advantages and limitations of this approach.

## 2 Using Shiny to Deploy a Web App for the Animal Culture Database

**FIGURE 1:**
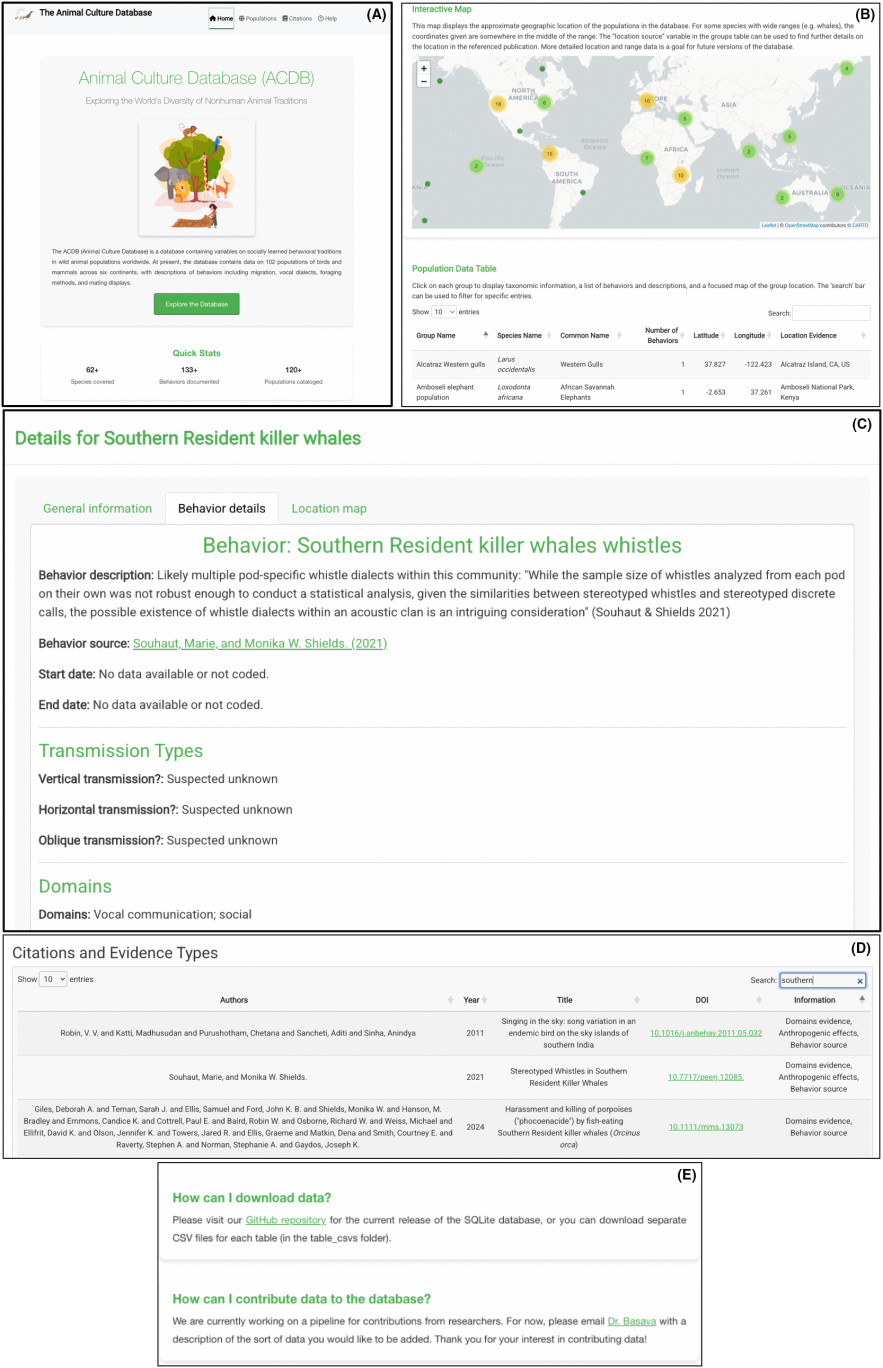
(A) Landing page of the Animal Culture Database (ACDB) web app. (B) Populations tab in the ACDB web app, showing a map summarizing occurrences and a table with additional information for each group. (C) Screenshot of the modals associated with one of the populations in the database. (D) Details of the Citations tab in the web app, including information on the data extracted from each cited paper. (E) Current version of the Help page in the web app, providing details on alternative sources, data retrieval, and other relevant information.

The Shiny app introduced here provides a user‐friendly interface to explore and analyze the current version of the Animal Culture Database (ACDB; Figure 1). The ACDB's web app enables users to efficiently navigate multiple datasets that summarize culturally transmitted behaviors across nonhuman animal populations worldwide. The ACDB is structured relationally, meaning that it consists of multiple linked tables, each representing different facets of information contained in the database. These tables are (1) species, which contains species‐level data including taxonomic information; (2) groups, which contains data on specific animal populations to which learned behavioral traditions have been attributed; (3) behaviors, which contains data on each behavior recorded for a population; and (4) sources, which contains the references used to provide evidence for records in each of the other tables. Further background on animal culture and motivations for building the database, along with definitions for each variable in the four tables, methods for construction, citations for included papers, current limitations, and goals for expansion are in Basava et al. (2025). Below, we briefly provide an overview of the web app's structure and relevant technical details of its implementation, including how data in each table is displayed through pages and tabs. The structured design of the ACDB web app ensures comprehensive access to the database while maintaining an intuitive and user‐friendly experience.

– Landing page: The landing page of the web app summarizes the current structure of the database by providing users with summary statistics on the number of groups, species, and behaviors included in the current release. This overview allows users to quickly review the scope and content of the ACDB.
– Populations tab: In this tab, users can explore a comprehensive list of groups exhibiting culturally relevant behaviors as recorded in the ‘groups’ table in the database. Information about populations is presented in two ways: (1) Interactive map: A map graphically illustrates the database's structure, rendered using leaflet (Cheng et al. 2024) with layers from OpenStreetMap (Haklay and Weber 2008). This map allows users to visualize the geographical distribution of culturally transmitted behaviors across populations. (2) Searchable table: A searchable table, generated using the DT R library (Xie et al. 2024), provides detailed information on each population. Each row displays taxonomic data, geographic coordinates, and the number of culturally transmitted behaviors documented. Users can click on a population either on the map or in the table to expand the view and reveal more detailed information. Due to intrinsic limitations in opening new tabs from within deployed Shiny apps, additional details for selected rows are displayed using modals within the same Shiny window (Chang et al. 2024). First modal: Displays general taxonomic information (from genus to phylum), conservation status from the IUCN, and the relevant social unit, which are in the ‘species’ table of the ACDB. The species GBIF taxonomic key is also displayed and links to the organism page on gbif.org. Second modal: Summarizes observed culturally transmitted behaviors as documented in the ‘behaviors’ table, categorized by behavioral domains (e.g., foraging, vocal communication, migration). This modal also lists descriptions and evidence of social transmission along with observations linking each behavior to potential conservation implications. Citations presented in each of the first two modals are linked to original publications via DOIs. This functionality enables users to directly access the source articles in new tabs outside of the web app. Third modal: Allows users to explore the geographical location of the recorded population in greater detail, providing spatial context for the behaviors documented.
– Other tabs: The web app includes additional tables (1) Citations tab: Lists all references used in the ACDB derived from the sources table, detailing the type of information extracted and its use within the database. This tab ensures transparency and proper attribution for all source material. It should be emphasized that the ACDB is a work in progress and this first version has sampled only a small portion of the total literature on animal behavioral traditions across wild populations (~1000 papers). Further details on the methodology for constructing the database via a systematic literature review and criteria for inclusion of studies according to a broad definition of culture can be found in Basava et al. (2025). (2) Frequently Asked Questions (FAQ) Tab: Provides details on accessing the source code for the web app, downloading the latest release of the database, and finding additional literature related to the ACDB.

## 3 Extending the ACDB Web App to Other Datasets

All the code and relevant files needed to fully run the latest release of the ACDB are in the following repository: https://github.com/datadiversitylab/ACDB. A simplified and annotated version of this Shiny app, with placeholders for text, images, tables, supporting tables, or relational databases as inputs, among other basic features, is available on GitHub: https://github.com/datadiversitylab/generic_shiny_data. Researchers interested in modifying the Shiny app can fork the repository and implement incremental changes to the app in accordance with their needs and structure of their data. The ACDB uses a relational database structure to present information on multiple aspects related to animal culture. The Shiny app reads the most recent version of a SQLite database into the server. This database is stored in the relevant GitHub repository, from which the Shiny app is deployed.db_path <- "db/ACDB_dev.sql"conn <- dbConnect(RSQLite::SQLite(), dbname = db_path)


The simplified template of the Shiny app supports both CSV files as inputs as well as relational databases. The sample dataset in this template is constructed based on the Palmer Penguins dataset (Gorman et al. 2014; Horst et al. 2020) instead of data from the ACDB. In the ACDB web app, multiple tables are stored within the database. In that case, we explicitly define the groups, species, behaviors, and sources tables. These objects are used downstream to generate maps and tables summarizing the information in the relational database. In the case of the template app, only a single table is available but more complex structures can be seen in the ACDB web app.groups_table <- dbReadTable(conn, "groups")species_table <- dbReadTable(conn, "species")behaviors_table <- dbReadTable(conn, "behaviors")sources_table <- dbReadTable(conn, "sources")


Users can follow the structure of the ACDB to replace the database, calls to the tables, and information presented in modals with their own datasets. We recommend starting from the simplified version of the web app to make these changes. Relational databases are not a strict requirement for the web app, as tables can be read in as CSV files (or other formats) depending on the developer's needs. Researchers interested in modifying the template or the ACDB web app should primarily focus on the server side of the app, as most critical elements are integrated into this component rather than the UI file.

## 4 Advantages and Limitations of Deployment Databases as Web Apps

The web app for the ACDB offers significant advantages but also presents notable limitations that relate to web apps more broadly. One limitation is the logistical and financial constraints associated with maintaining an active web‐based platform. Although deploying the ACDB as a Shiny app enhances accessibility (e.g., Ronquillo et al. 2024), it requires continuous server resources and incurs hosting costs that may increase with user demand (Shryock et al. 2023). Platforms like ShinyApps.io provide free tiers, but their limited computational power and bandwidth could restrict concurrent access. Self‐hosting through institutional or private servers addresses some of these issues but requires advanced technical expertise and ongoing maintenance. Additionally, sustaining the web app over time poses challenges, as web applications require continuous updates to remain compatible with evolving software dependencies and system requirements. Despite these limitations, deploying the ACDB as a Shiny app offers several key advantages. Shiny's integration within the R programming environment enables researchers to transform static datasets into interactive platforms without requiring extensive web development expertise. Unlike other web frameworks, Shiny does not necessitate proficiency in JavaScript, HTML, or backend services, making it accessible to researchers with minimal coding experience. Furthermore, the structured framework used in this deployment offers a scalable and standardized approach for releasing tabular data, ensuring that users can easily query, visualize, and download information without complex data wrangling.

## Author Contributions

**Kiran Basava:** conceptualization (equal), data curation (lead), writing – original draft (equal), writing – review and editing (equal). **Cristian Román‐Palacios:** conceptualization (equal), software (lead), writing – original draft (equal), writing – review and editing (equal).

## Conflicts of Interest

The authors declare no conflicts of interest.

## Data Availability

The ACDB GitHub repository can be found at https://github.com/datadiversitylab/ACDB. This includes the SQLite database and each table as a CSV file. The deployed site is currently under https://datadiversitylab.github.io/ACDB/. Code and relevant files needed to run the latest release of the ACDB are in https://github.com/datadiversitylab/ACDB_datarelease. The simplified and generic Shiny app associated with the ACDB can be found at: https://github.com/datadiversitylab/generic_shiny_data.
